# Ionic Liquids: A Simple Model to Predict Ion Conductivity Based on DFT Derived Physical Parameters

**DOI:** 10.3389/fchem.2019.00126

**Published:** 2019-03-12

**Authors:** Jonathan Nilsson-Hallén, Bodil Ahlström, Maciej Marczewski, Patrik Johansson

**Affiliations:** Department of Physics, Chalmers University of Technology, Gothenburg, Sweden

**Keywords:** ionic liquid, conductivity, DFT, model, ion

## Abstract

A model able to a priori predict ion conductivities of ionic liquids (ILs) is a desired design tool. We here propose a set of simple conductivity models for ILs composed of small ions by only using data easily derived from standard DFT calculations as input; ion volume, ion mass, ion moment of inertia, and the ion-ion interaction strength. Hence these simple models are totally without any need for experimental parametrization. All model are made from fits of 22 ILs based on 12 different cations and 5 different anions, resulting in correlations vs. experiment of *R*^2^≈0.95 and MAE of 25–36%. Given their (very) simple layout and how fast they can be applied (and re-used), the models allow for ample screening of new IL designs, while not aimed for perfect predictions *per se*.

## Introduction

Ionic liquids (ILs) represent an interesting class of materials that during the last few decades have been intensely studied (Plechkova and Seddon, 2008; Castner and Wishart, 2010; Thematic issue: Physical chemistry of ionic liquids, 2010; Thematic issue: Ionic liquids, 2012), partly driven by the many potential applications suggested, such as electrolytes in electrochemical energy storage and conversion devices e.g., batteries, fuel cells, solar cells, etc (Armand et al., 2009; Lewandowski and Swiderska-Mocek, 2009; Thematic issue: Ionic liquids for energy applications, 2013). Due to the large number of possible ILs, often estimated to 10^12^-10^14^, or even up to 10^18^(!) (Plechkova and Seddon, 2008), methods for predicting the properties of ILs *a priori* to any time-consuming synthesis attempts are highly desirable. Any such method needs to take stance at the ion level and should preferably be general, accurate, non-empirical, and fast. Indeed, there are many different standard modeling approaches applied to ILs from *ab initio*, DFT, MD, to *ab initio* molecular dynamics (AIMD) (Del Pópolo et al., 2005; Tsuzuki et al., 2005; Borodin, 2009; Kirchner, 2009; Maginn, 2009; Angenendt and Johansson, 2010; Johansson et al., 2010; Ueno et al., 2010; Liu and Maginn, 2012; Tsuzuki, 2012), and more analytical methods (Abbott, 2004, 2005; Matsuda et al., 2007; Slattery et al., 2007; Tochigi and Yamamoto, 2007; Preiss et al., 2010; Eiden et al., 2011). All aim at predicting/estimating melting points (Slattery et al., 2007; Preiss et al., 2010), viscosities (Abbott, 2004; Matsuda et al., 2007; Slattery et al., 2007; Tochigi and Yamamoto, 2007; Ueno et al., 2010; Eiden et al., 2011), and not the least the ionic conductivities (Abbott, 2005; Del Pópolo et al., 2005; Tsuzuki et al., 2005; Matsuda et al., 2007; Slattery et al., 2007; Tochigi and Yamamoto, 2007; Borodin, 2009; Johansson et al., 2010; Ueno et al., 2010; Eiden et al., 2011; Liu and Maginn, 2012; Tsuzuki, 2012). While in general highly successful for their purposes, they often, however, have either limited accuracy, are time-consuming or require ion-specific or empirical parameters.

From an experimental point of view ion conductivity data are most often provided as Arrhenius plots, σ = f(1/T), and can for ILs be accurately fitted using the Vogel-Tamman-Fulcher (VTF) equation (Vila et al., 2006),

(1) $$\sigma =\;\sigma_{\infty }\exp\left(-\frac{E_{a}}{k_{b}\left(T-T_{0}\right)}\;\right)$$

where σ_∞_ is the conductivity at infinite temperature, E_a_ is the activation energy, and T_0_ is a constant related to transformation of the observed glass transition temperature, T_g_, to the ideal.

The general features of the VTF equation, together with a lot of other types of expressions, were here used as starting points to generate models for the ionic conductivity of ILs. Many previous IL conductivity models have shown a strong dependency on the ion volumes, but require ion-specific, most often anion-specific, parameters (Tsuzuki et al., 2005; Matsuda et al., 2007; Slattery et al., 2007; Borodin, 2009; Angenendt and Johansson, 2010; Ueno et al., 2010; Tsuzuki, 2012) or resort to analyzing ILs with very similar anions (Johansson et al., 2010). In order to avoid this, which severely limits the generality, we have included not only the ion volumes, but also ion (reduced) masses—as proven to affect the viscosity (Abbott, 2004, 2005; Matsuda et al., 2007), and ion moments of inertia as input parameters to a wide variety of (VTF-based) models. Furthermore, as a combination of (at least) two ions, a cation and an anion, is needed to create any IL, the strength of the specific ion-ion interaction was used as another parameter—more or less as a proxy for the activation energy (E_a_) in the VTF expression—even if the connection between these is far from obvious (Tsuzuki et al., 2005; Borodin, 2009; Angenendt and Johansson, 2010; Johansson et al., 2010; Ueno et al., 2010; Tsuzuki, 2012). We would also like to stress that we here avoid any additional complexity of IL nano-structuration by only studying ILs with rather short cation alkyl chain lengths. We have also chosen to include imidazolium based ILs with the C2 position methylated as these seem to introduce a specific behavior in terms of ion-ion interaction. All the parameters are obtained starting from standard density functional theory (DFT) calculations on ions and cation-anion ion pairs—and can thus be used to create models totally without any experimental parametrization—one major advantage of the approach chosen.

The various models developed in this study have been fitted and evaluated against in-house measured ionic conductivities. While this clearly limits the statistics of the study in terms of quantity, it improves the quality as it avoids the errors that can result from gathering data from many different sources—important as reported IL conductivities are very sensitive to impurities, not the least water, and the set-up/sample history. From this approach the best fits are reported as largely phenomenological models using some few statistical measures and then discussed in terms of e.g., sensitivity vs. the different input parameters computed and the ILs chosen to study.

## Materials and Methods

In total 22 different ILs were used as received from Solvionic with the highest purity available (99.9 or 99.5%). The temperature dependent ionic conductivity was measured using a Novocontrol broadband dielectric spectrometer in the frequency range 10^−1^-10^7^ Hz. A liquid parallel plate cell, BDS 1308 Novocontrol, with two stainless steel electrodes with a Teflon spacer (Ø = 13.2 mm, d = 0.99 mm) was assembled in an Ar glove box (H_2_O and O_2_ < 1 ppm) and loaded into a cryo-furnace with N_2_ gas flow. Data were collected during heating from 298.15 to 368.15 K in steps of 10 K and stabilization times of 30 min. The DC conductivities were obtained from the low frequency plateau of the conductivity isotherms by plotting the real part of the complex conductivity as a function of frequency.

Computationally low-energy conformers of all cations (12) and anions (5) (Table 1) constituting the totally 22 ILs investigated experimentally were initially generated by the molecular mechanics based scan routine in Spartan 06^1^. Amongst the cations we use the most popular families: imidazolium, EMI etc., and pyrrolidinium, MPPyr (Pyr13) etc. From these the geometries and energies were optimized by DFT, first at the B3LYP/6-311+G^*^ level and subsequently further with M06-2X/6-311+G^*^ (Vosko et al., 1980; Lee et al., 1988; Becke, 1993; Zhao and Truhlar, 2007), and their 2nd derivatives calculated to ensure the geometries to be local minima energy structures. From the DFT results the ion volumes were calculated as averages of 10 runs using a Monte Carlo (MC) integration scheme of a 0.001 e/bohr^3^ density envelope and 100 integration points/bohr^3^. The moments of inertia of the ions were calculated as the arithmetic mean of the three principal eigenvalues obtained from the routine in the VMD software (Humphrey et al., 1996).

**Table 1:** Name (acronym), volume (V), mass (M), and moment of inertia (I) of ions.

**Name**	**V [Å^**3**^]**	**M [u]**	**I [Å^**2**^u]**
1-ethyl-3-methylimidazolium (EMI)	156	111	315
1-butyl-3-methylimidazolium (BMI)	196	139	577
1-hexyl-3-methylimidazolium (HMI)	246	167	1,041
1,3-dimethyl-2-propylimidazolium (PMMI)	204	139	536
1,3-dimethyl-2-butylimidazolium (BMMI)	224	153	760
trimethyl-butylammonium (Me_3_BuN)	111	193	429
trimethyl-hexylammonium (Me_3_HexN)	148	240	934
diethylmethylpropylammonium (Et_2_MeProN)	212	130	438
1-propyl-1-methylpyrrolidinium (MPPyr)	199	128	409
1-butyl-1-methylpyrrolidinium (BMPyr)	222	142	638
1-pentyl-1-methylpyrrolidinium (MPePyr)	242	156	896
1-propyl-1-methylpiperidinium (MPPip)	221	142	483
tetrafluoroborate (BF_4_)	71	87	95
hexafluorophosphate (PF_6_)	96	145	201
trifluoromethanesulfonate (Tf)	115	149	276
bis(fluorosulfonyl)imide (FSI)	136	180	446
bis(trifluoromethanesulfonyl)imide (TFSI)	199	280	1,214

The lowest energy conformers (B3LYP) for each of the ions were used to generate cation-anion ion pairs, with 20 starting configurations for each pair generated by our in-house developed software SECIL (Angenendt and Johansson). All structures were optimized in two steps; i) B3LYP/6-311+G^*^ - providing a selection of 10 structures/ion pair based on energy and transferred to ii) M06-2X/6-311+G^*^. Not all SECIL generated configurations reach SCF convergence, but most B3LYP minima produce M06-2X minima, as verified by the 2nd derivatives. All DFT and MC calculations were made using the Gaussian 03 and 09 program packages (Frisch et al., 2004, 2010). From the M06-2X/6-311+G^*^ data the ion-ion interaction energy, E_int_ = E_(ion pair)_-*Σ*(E_cation_+E_anion_), was calculated—as a measure of specific ion-ion interaction strength (Table 2). Due to the main aim of arriving at a proof-of-concept method, and not the most performant, and the many other model omissions and simplifications, no comparisons to other DFT functionals (or higher *ab initio* methods) nor any basis set size effect investigations or basis set super-position error corrections were made.

**Table 2:** Cation-anion ion-pair, E_int_, and glass transition temperature (T_g_) of the ILs.

**Cation-Anion**	E_**int**_ (M06-2X/6-311+G*)[kJmol^**−1**^]	**T_**g**_[K]**
EMI-Tf	−378.9	–
EMI-FSI	−362.3	–
EMI-TFSI	−360.5	181 (Fredlake et al., 2004)
BMI-BF_4_	−385.0	188 (Fredlake et al., 2004)
BMI-PF_6_	−363.6	197 (Fredlake et al., 2004)
BMI-Tf	−376.5	191 (Tokuda et al., 2006)
BMI-TFSI	−367.3	187 (Fredlake et al., 2004)
HMI-PF_6_	−362.2	195 (Harris et al., 2007)
HMI-Tf	−374.6	189 (Gómez et al., 2013)
HMI-TFSI	−363.7	189 (Gómez et al., 2013)
PMMI-TFSI	−364.2	–
BMMI-TFSI	−363.7	–
Me_3_BuN-TFSI	−343.9	199 (Tokuda et al., 2006)
Me_3_HexN-TFSI	−353.5	202 (Taggougui et al., 2008)
Et_2_MeProN-FSI	−340.7	–
MPPyr-FSI	−343.3	190 (Zhou et al., 2008)
MPPyr-TFSI	−346.8	188 (Calvar et al., 2013)
BMPyr-Tf	−361.5	197 (Calvar et al., 2013)
BMPyr-TFSI	−351.2	191 (Calvar et al., 2013)
MPePyr-TFSI	−352.1	–
MPPip-FSI	−332.9	–
MPPip-TFSI	−338.5	192 (Paduszynski et al., 2011)

Using the ion volume (V), mass (M), moment of inertia (I) and ion pair (E_int_, reduced mass) data generated, potential models for the conductivity as function of temperature were formulated and tested with OriginPro 2015^2^, using the Levenberg-Marquardt iteration algorithm to fit the data to a predefined function. The most promising models were further combined to achieve better fits, while kept physically sound.

## Results and Discussion

**Figure 1:**
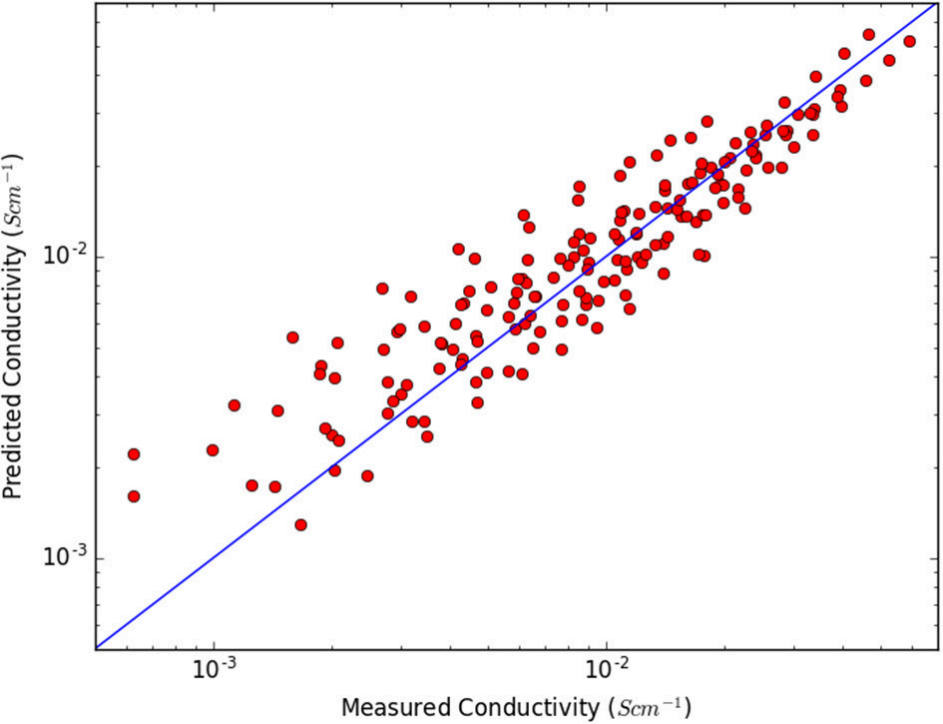
Measured and predicted conductivities using model A.

The computational data generated span an appreciable range of IL ion volumes (71–246 Å^3^), masses (87–280 u), moments of inertia (95–1214 Å^2^u), and interaction energies (−333 to −385 kJmol^−1^). The first model found with an appreciable fit to the conductivity data, 176 data points for the 22 ILs, is **A** (Figure 1):

(A) $$\sigma =\frac{b}{V_{tot}{}^{2}I_{+}}\exp\left(c\frac{Eint}{T-T_{0}}\right)$$

where the input parameters *V*_*tot*_ is the sum of the cation and anion volumes, *I*_+_ is the moment of inertia of the cation, and *E*_*int*_ is the interaction energy, while *b, c*, and *T*_0_ are fitting parameters. That the conductivity is inversely proportional to the ion volumes, as in **A**, can easily be understood as larger ions both move slower and hinder movement of other ions and this has indeed been found previously (Slattery et al., 2007; Johansson et al., 2010; Tsuzuki, 2012), but why it should be proportional to the inverse of the volume squared is unclear.

**Figure 2:**
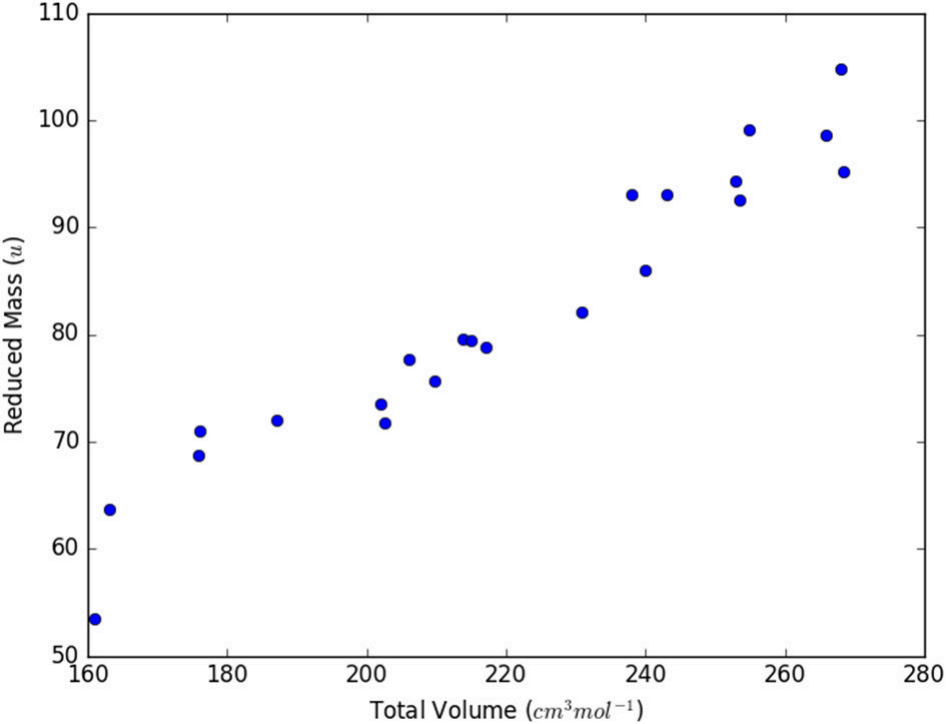
Correlation between total volume and reduced mass for the IL ion pairs.

**Figure 3:**
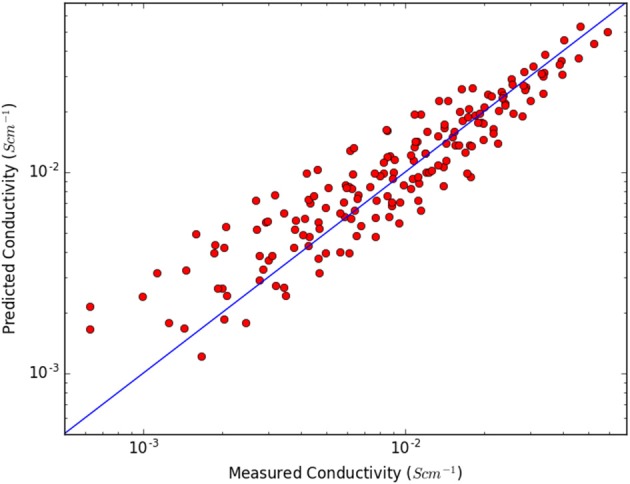
Measured and predicted conductivities using model **A2**.

It has previously been shown that the viscosity of ILs is affected by the IL ion masses (Abbott, 2004, 2005; Matsuda et al., 2007) and as the conductivity is related to the fluidity, the inverse of the viscosity through the fractional Walden rule, it is reasonable to assume that these could affect the conductivity. The reduced masses, M_red_, are indeed almost linearly proportional to the volumes V_tot_ (Figure 2).

why a second model, **A2**, with one of the terms exchanged, was investigated—with almost equally good fit (Figure 3):

(A2) $$\sigma =\frac{b}{V_{tot}M_{red}I_{+}}\exp\left(c\frac{Eint}{T-T_{0}}\right)$$

In both models **A** and **A2** T_0_ has to some extent been treated as a normal fitting parameter. This enables a true prediction of conductivity without any prior IL synthesis, but given the coupling of T_0_ to T_g_, experimental T_g_ data as input might improve the fits and models. In the literature T_g_ for 15 of the 22 ILs used were found (Table 2) covering the range 181-202 K (which arguably is a rather limited span in temperature). Applying these data to **A** and **A2** result in models **B** and **B2**, respectively:

(B) $$\sigma =\frac{b}{V_{tot}{}^{2}I_{+}}\exp\left(c\frac{Eint}{T-(T_{g}+d)}\right)$$

(B2) $$\sigma =\frac{b}{V_{tot}M_{red}I_{+}}\exp\left(c\frac{Eint}{T-(T_{g}+d)}\right)$$

where *d* is an additional fitting parameter and indeed the fits are slightly improved. Using the reduced data set (15/22) in **A** and **A2** did not improve their fits and thus the improvement indeed comes from the inclusion of T_g_ (keeping in mind the limited T range). All models with their parameter values and statistical measures are summarized in Table 3.

**Table 3:** Fitting parameter values, correlation coefficients (R^2^), and mean absolute errors (MAE) for the four IL conductivity models.

**Model**	**b [Scm^**−1**^Å^**8**^u]**	**c [KkJ^**−1**^]**	**T_**0**_/d [K]**	***R*^**2**^**	**MAE [%]**
A	1.29 ± 0.45 × 10^7^	−1.01 ± 0.26	204 ± 18.0	0.9538	35.3
A2	2.52 ± 0.88 × 10^6^	−0.90 ± 0.25	211 ± 18.0	0.9508	36.5
B	1.09 ± 0.38 × 10^7^	−0.82 ± 0.23	34.6 ± 15.9	0.9627	24.8
B2	3.19 ± 1.22 × 10^6^	−1.01 ± 0.28	22.6 ± 17.6	0.9600	26.1

## Concluding Remarks

Two VFT based models for IL conductivity without any experimental input have been developed using volume, mass, and moment of inertia of the constituting ions and the energy of the interaction between cation and anion. In contrast to earlier models there are no anion-specific parameters needed, and in addition moments of inertia of the cations are for the first time included. For the latter feature we initially believed a larger principal moment of inertia to be likely to have a decisive influence due to the elongated shape of many of the ions. However, no such behavior was found in the fits why the arithmetic mean of the three principal values is used. The main influence by the cation moment of inertia is due to many anions being close to spherical. All models without *I*_+_ performed much worse than the fits here obtained and the inclusion of experimental glass transition temperatures improves the fit, but not to any large extent. From a physical point of view, the ion mass may influence the conductivity, why both A2 and B2, replacing one of the volume terms with the reduced masses of the ion pairs, could improve the fit if applied to a larger and more diverge data set (even if they here actually reduce the goodness of the fit). As VFT models in general captures ion conductivities of ILs (and other systems) quite well all of the above in terms of overall agreement come as no major surprise. Phenomenonlogically all the four (VFT) models show the expected behavior; as the volumes, moments of inertia, and masses of the ions increase, the conductivity will approach zero (0), and if they decrease the conductivity will increase. Likewise, if the ion-ion interaction energy increases to infinity the conductivity will approach zero and the conductivity increases with temperature. The inclusion of experimental glass transition temperatures did improve the fit, but there is a rather narrow temperature range for the T_g_ used. We stress that we do refrain from trying to connect the input parameters to any direct physical interpretation—such as why the prefactor in the VFT equations seem to relate to the ion volume squared, and how in the exponential the ion-ion interaction energy relate to the activation energy, etc. We do, however, believe it to be straightforward to produce input to these models for any IL of choice—remembering our deliberatively set limits of no nano-structured ILs—for which this model is likely to fail—dependent on how the ion mobility is affected. The DFT calculations will be the time-determining step, even if most data required only needs to be calculated once for each ion, the interaction energy of each ion pair must be obtained separately for each IL.

## Author Contributions

JN-H performed the fits based on input from PJ and BA and made a first draft of the paper. MM measured all experimental data. PJ outlined the idea and wrote the final manuscript.

### Conflict of Interest Statement

The authors declare that the research was conducted in the absence of any commercial or financial relationships that could be construed as a potential conflict of interest.

## Footnotes

^1^Spartan '06, Wavefunction Inc.

^2^OriginLab, Northampton, MA, USA
